# Supplementary material for: PEPhub: a database, web interface, and API for editing, sharing, and validating biological sample metadata
Source: bioRxiv. 2024 May 11:2023.08.15.551388. Originally published 2023 Aug 18. Preprint. [Version 2] doi: 10.1101/2023.08.15.551388 (PMC10462087; doi:10.1101/2023.08.15.551388)
Supplement: Supplement 1 [file NIHPP2023.08.15.551388v2-supplement-1.pdf]

bioRxiv preprint doi: https://doi.org/10.1101/2023.08.15.551388; this version posted May 11, 2024. The copyright holder for this preprint (which was not certified by peer review) is the author/funder, who has granted bioRxiv a license to display the preprint in perpetuity. It is made available under aCC-BY 4.0 International license.

Supplemental figures

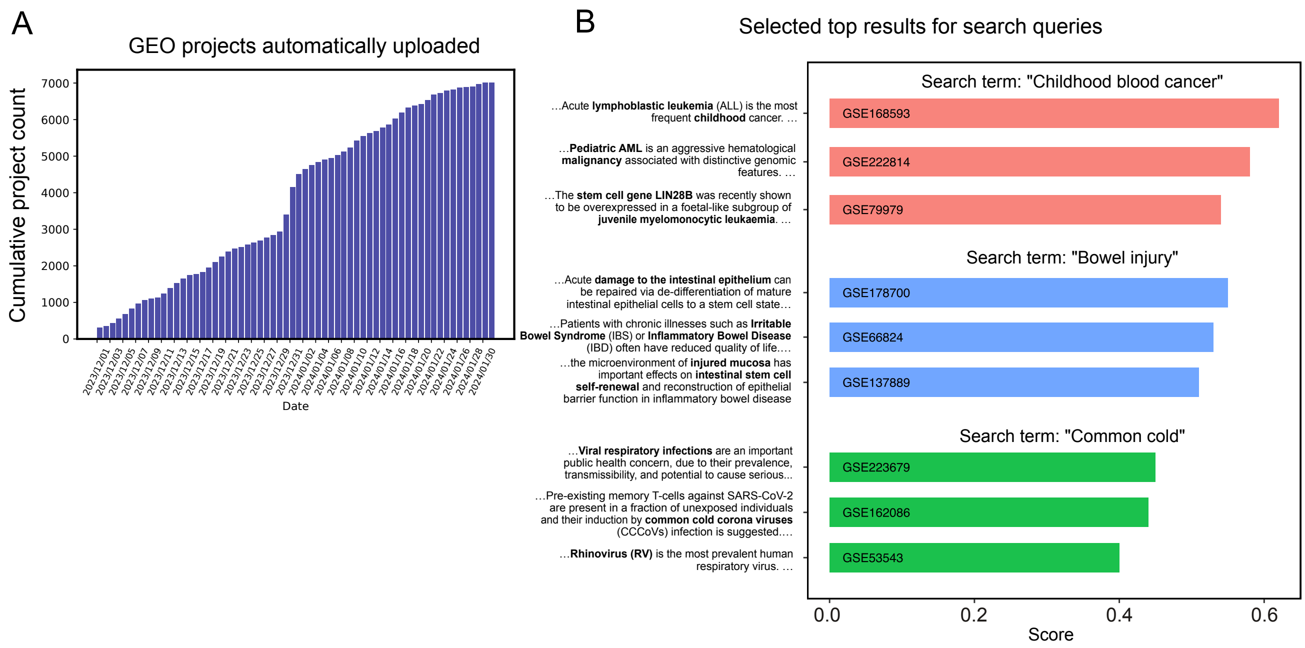

Supplementary Figure S1. Analysis of PEP metadata and search. *A) Barchart showing the cumulative number of new biological sample tables added to PEPhub from GEO, automatically. PEPhub automatically indexed more than 1000 new projects during this 3-week demo span. B) Illustrative search result scores selected from the top 10 responses returned by the PEPhub semantic search engine for biological search terms shown*

Supplemental text

Search result examples

Illustrative search results from PEPhub. Search results from GEO and PEPhub for common queries. The PEPhub search engine returns results that are more relevant to the query than the GEO search engine. Moreover, the results returned by PEPhub are more diverse.

Search term: “Childhood blood cancer”

GEO results:

- . . . effects of MZ1 on multiple molecular subtypes of B-cell acute lymphoblastic leukemia cells (GSE217540)
- . . . PAF1 and FACT to drive high density enhancer interactions in leukemia (GSE202451)
- . . . PAF1 and FACT to drive high density enhancer interactions in leukemia. . . (GSE202450)
- . . . PAF1 and FACT to drive high density enhancer interactions in leukemia. . . (GSE202449)

PEPhub results:

- . . . genomic alterations in radiation-related breast cancer among childhood cancer survivors. . . (GSE62940)
- . . . analysis of CD10+/CD19+ pre B lymphoblasts from bone marrow and peripheral blood of B-ALL patients (GSE168593)
- . . . DNA methylation profiling predicts relapse in childhood B-cell acute lymphoblastic leukemia (GSE39141)
- . . . analysis of pediatric histiocytic sarcomas and anteceding hematologic malignancies (GSE109904)

Search term: “Bowel injury”

GEO results:

- tumors from mice fed diets excluding methionine/tryptophan/niacin (GSE246627)
- tumors from mice fed diets excluding methionine/tryptophan/niacin (GSE246626)
- Airway Microfold (M) Cells Emerge in the Post-IAV Lung (GSE244279)

PEPhub results:

- Colonic mucosal injury responses (GSE164918)

bioRxiv preprint doi: https://doi.org/10.1101/2023.08.15.551388; this version posted May 11, 2024. The copyright holder for this preprint (which was not certified by peer review) is the author/funder, who has granted bioRxiv a license to display the preprint in perpetuity. It is made available under aCC-BY 4.0 International license.

Search term: “Common cold”

GEO results:

- ATAC-Seq of Batf-deficient pDC Transcriptomes… (GSE178410)
- Deterministic reprogramming of neutrophils in tumors (GSE244536)
- …Pancreatic Tumors Reveal Distinct Compartmentalisation of Neutrophil Subsets (GSE244534)
- …of neutrophil subsets in a mouse model of pancreatic cancer (GSE244531)

PEPhub results:

- A longitudinal study of natural respiratory viral infections (GSE223679)
- …temperature variation controls pre-mRNA processing and transcription of anti-viral genes (GSE193639)
- Influenzavirus serotype association to global whole blood transcriptional changes (GSE29385)
- The immune response and microbiota profiles during co-infection with P. vivax… (GSE144792)
