# Supplementary material for: PEPhub: a database, web interface, and API for editing, sharing, and validating biological sample metadata
Source: bioRxiv. 2023 Aug 18:2023.08.15.551388. Preprint. [Version 1] doi: 10.1101/2023.08.15.551388 (PMC10462087; doi:10.1101/2023.08.15.551388)
Supplement: Supplementary file 1 [file NIHPP2023.08.15.551388v1-supplement-1.pdf]

bioRxiv preprint doi: https://doi.org/10.1101/2023.08.15.551388; this version posted August 18, 2023. The copyright holder for this preprint (which was not certified by peer review) is the author/funder, who has granted bioRxiv a license to display the preprint in perpetuity. It is made available under aCC-BY 4.0 International license.

Supplemental figures

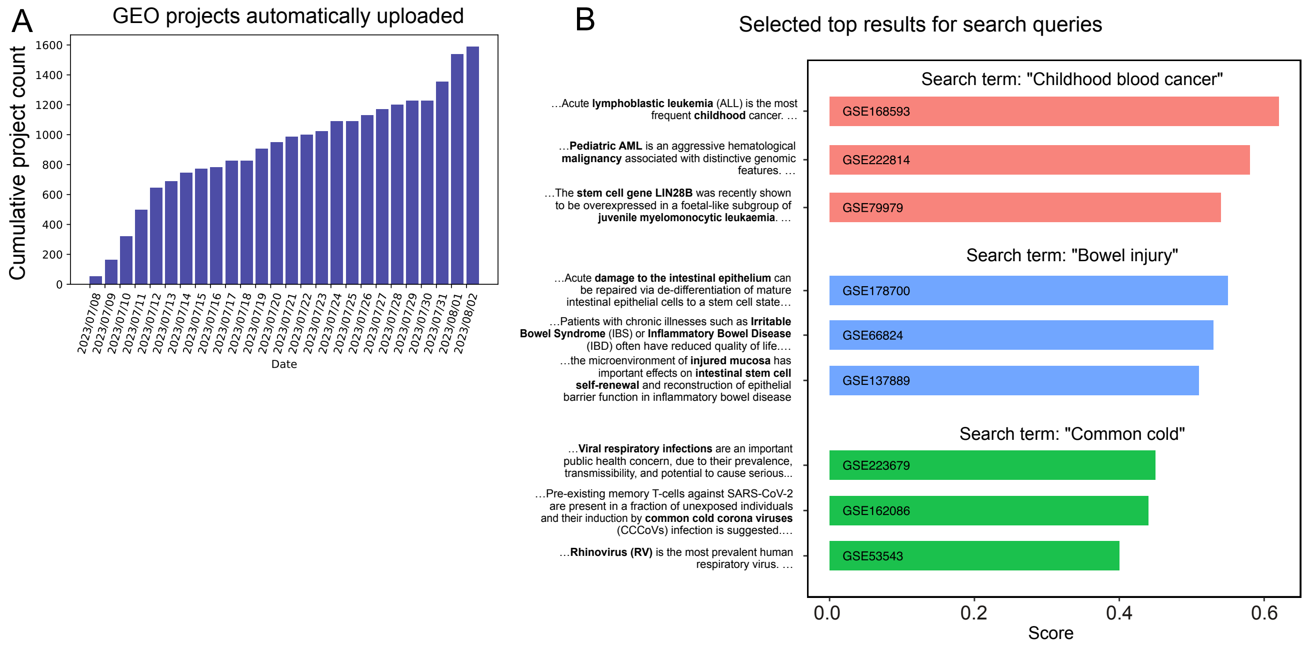

Supplementary Figure S1. Analysis of PEP metadata and search. *A) Barchart showing the cumulative number of new biological sample tables added to PEPhub from GEO, automatically. PEPhub automatically indexed more than 1000 new projects during this 3-week demo span. B) Illustrative search result scores selected from the top 10 responses returned by the PEPhub semantic search engine for biological search terms shown*
